# Impact of Breast Cancer Early Detection Training on Rwandan Health Workers’ Knowledge and Skills

**DOI:** 10.1200/JGO.17.00098

**Published:** 2018-01-25

**Authors:** Lydia E. Pace, Jean-Marie Vianney Dusengimana, Nancy L. Keating, Vedaste Hategekimana, Vestine Rugema, Jean Bosco Bigirimana, Ainhoa Costas-Chavarri, Aline Umwizera, Paul H. Park, Lawrence N. Shulman, Tharcisse Mpunga

**Affiliations:** **Lydia E. Pace**, **Nancy L. Keating**, and **Paul H. Park**, Brigham and Women’s Hospital and Harvard Medical School, Boston, MA; **Jean-Marie Vianney Dusengimana**, **Jean Bosco Bigirimana**, and **Paul H. Park**, Partners in Health/Inshuti Mu Buzima; **Ainhoa Costas-Chavarri**, Rwanda Military Hospital, Kigali; **Vedaste Hategekimana**, **Vestine Rugema**, **Aline Umwizera**, and **Tharcisse Mpunga**, Ministry of Health, Butaro, Rwanda; and **Lawrence N. Shulman**, Abramson Cancer Center, University of Pennsylvania, Philadelphia, PA.

## Abstract

**Purpose:**

In April 2015, we initiated a training program to facilitate earlier diagnosis of breast cancer among women with breast symptoms in rural Rwanda. The goal of this study was to assess the impact of the training intervention in breast cancer detection on knowledge and skills among health center nurses and community health workers (CHWs).

**Methods:**

We assessed nurses’ and CHWs’ knowledge about breast cancer risk factors, signs and symptoms, and treatability through a written test administered immediately before, immediately after, and 3 months after trainings. We assessed nurses’ skills in clinical breast examination immediately before and after trainings and then during ongoing mentorship by a nurse midwife. We also examined the appropriateness of referrals made to the hospital by health center nurses.

**Results:**

Nurses’ and CHWs’ written test scores improved substantially after the trainings (overall percentage correct increased from 73.9% to 91.3% among nurses and from 75.0% to 93.8% among CHWs (*P* < .001 for both), and this improvement was sustained 3 months after the trainings. On checklists that assessed skills, nurses’ median percentage of actions performed correctly was 24% before the training. Nurses’ skills improved significantly after the training and were maintained during the mentorship period (the median score was 88% after training and during mentorship; *P* < .001). In total, 96.1% of patients seen for breast concerns at the project’s hospital-based clinic were deemed to have been appropriately referred.

**Conclusion:**

Nurses and CHWs demonstrated substantially improved knowledge about breast cancer and skills in evaluating and managing breast concerns after brief trainings. With adequate training, mentorship, and established care delivery and referral systems, primary health care providers in sub-Saharan Africa can play a critical role in earlier detection of breast cancer.

## INTRODUCTION

The burden of breast cancer is rising in low- and middle-income countries (LMICs), where mortality-to-incidence ratios are high because of advanced stage at presentation and limited access to effective treatment.^1^ Long diagnostic delays seem to be a major contributor to late-stage presentations.^2^ Optimal ways to promote earlier detection of breast cancer in LMICs are not yet known, because mammography screening is not yet widely available in most low-income settings.^1^ Experts increasingly recommend focusing on clinically detectable disease and building robust referral, diagnostic, and treatment strategies before population-based screening of asymptomatic women.^3^ Evaluating effective and feasible strategies in LMICs to facilitate earlier breast cancer detection is a global health priority.^4^

Rwanda is a low-income, predominantly rural country of 12 million people in East Africa. In research conducted at Butaro Cancer Center of Excellence (BCCOE), a rural Rwandan public cancer facility, we found that women experienced median delays of 15 months between the onset of breast symptoms and receipt of a breast cancer diagnosis.^5^ Patient delays (between symptom onset and first presentation at a health facility) and system delays (between first presentation at a health facility and ultimate diagnosis) were equally long, and patient or system delays of more than 6 months were associated with greater likelihood of having metastatic disease at diagnosis.^5^ More than half the patients had visited a health facility five or more times for their breast symptoms before receiving a cancer diagnosis.

In response to these findings, we implemented a pilot early-detection program in Burera District, the rural district of approximately 340,000 people where BCCOE is located. The foundation of Rwanda’s health care system consists of rural health centers staffed by nurses and lay community health workers (CHWs) who play many roles, including building community awareness about preventive health care and connecting individuals with the health care system. We sought to address patient delays by training CHWs to educate community members about signs and symptoms of breast cancer and encourage them to seek evaluation for breast concerns. To address system delays, we trained health center nurses in the evaluation of breast concerns, established weekly health center breast clinics, and provided sustained on-site mentorship. We also established a hospital-based breast clinic to provide efficient evaluation and management for referred patients. We sought to determine the value and feasibility of such a training program by assessing its impact on health workers’ knowledge and skills, patient volume and service delivery, and patients’ experiences and outcomes. Here we report the impact of the training on nurses’ and CHWs’ knowledge and skills.

## METHODS

### Intervention

**Fig 1:**
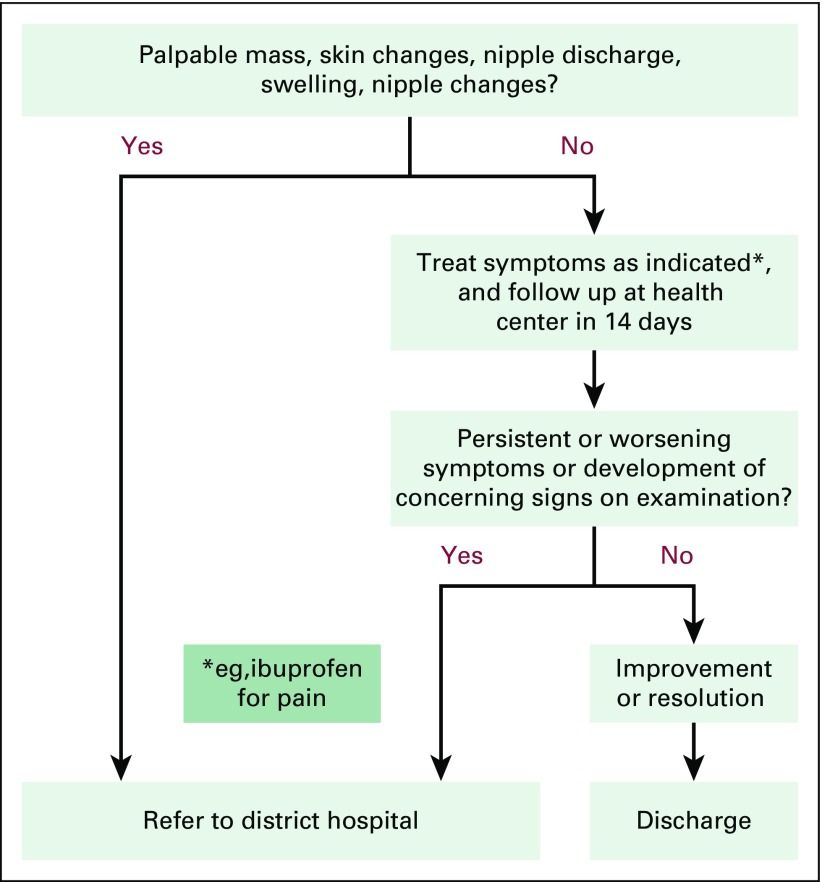
Algorithm for management of breast concerns among non-breastfeeding women at the health center level.

**Fig 2:**
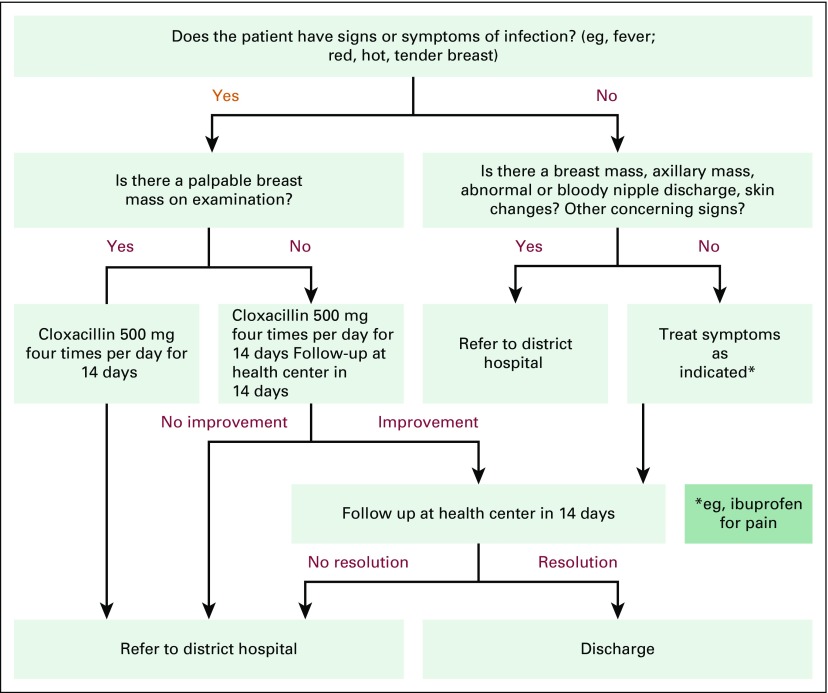
Algorithm for management of breast concerns among breastfeeding women at the health center level.

The training intervention took place in two stages. In the first stage, we randomly selected seven of the 19 health centers in Burera District, along with their affiliated CHWs, to receive trainings in April and May 2015. In the second stage, we randomly assigned an additional five health centers and affiliated CHWs to receive trainings in November and December 2015. The remaining seven health centers in Burera District served as controls. Before the start of trainings, we identified a group of BCCOE clinicians with extensive experience in the evaluation of breast disease to become trainers. We then held a 1-day training-of-trainers during which US and Rwanda-based clinicians experienced in breast oncology and surgery familiarized the trainers with the training materials, standards for high-quality clinical breast examination (CBE), and teaching and evaluation techniques. Trainings for health center nurses lasted 3 to 4 days and included (1) didactic teaching on signs and symptoms of benign and malignant breast disease, cancer risk factors, and basics of breast cancer diagnosis and treatment; (2) practical teaching in performing CBE using breast models (Gaumard Breast Palpation Simulators); and (3) training in two algorithms that address evaluation and management of specific breast concerns (one in breastfeeding women and one in non-breastfeeding women; Figs 1 and 2) that included when patients should be referred to the hospital. Nurses were also instructed in training CHWs. After this initial training, health centers were supported in establishing weekly breast clinics for patients with breast concerns. They were provided with documentation forms to use and were asked to refer patients to the BCCOE breast clinic as needed. A hospital-based nurse-midwife trained as a breast health mentor visited each of these health center breast clinics every 1 to 2 weeks to provide clinical supervision and mentorship of the trained nurses; nurses were also encouraged to call her with questions.

The training intervention for CHWs consisted of a 1-day didactic session led by trained health center nurses and their BCCOE-based clinician trainers. All CHWs who worked in each intervention health center’s catchment area were invited to participate; this included Animatrice de Santé Maternelle CHWs tasked with maternal and infant health and Agent de Santé Binome (Binome) CHWs who worked with community members on issues related to HIV, tuberculosis, malaria, malnutrition, and prevention. CHWs were taught about the signs, symptoms, and risk factors associated with breast cancer and the availability of affordable breast cancer treatment in Rwanda. They were then asked to lead educational sessions with their villages and encourage individual women with breast symptoms to seek prompt evaluation at health centers.

### Evaluation of Knowledge and Skills

Nurses’ and CHWs’ knowledge was assessed through written tests administered immediately before and immediately after trainings, and delayed post-tests administered 3 to 6 months after training. Questions were developed through review of existing instruments used in the United States,^6,7^ Mexico,^8^ the United Kingdom,^9^ and Nigeria,^10^ and expert input, feedback from clinical colleagues in Rwanda, and pilot testing with Rwandan hospital-based nurses. We assessed each nurse’s and CHW’s overall test score as the total percentage of questions correct and also assessed scores within specific knowledge domains. Nurses’ skills in history-taking, CBE, patient counseling, and management decisions were assessed by using standardized checklists during observation of role play and CBE on breast models immediately before and after the training. CBE materials were based on CBE guidelines^11,12^ and published^13^ and unpublished^3^ curricula adapted to our setting. Subsequently, during her regular health center visits, the breast health mentor assessed nurses’ clinical skills in their clinics by using a checklist that included the same questions as the pre- and post-test assessment form. When possible, the mentor assessed individual nurses by using the same checklist two or more times during a given clinical session, including one at the beginning of a session. However, we did not document which checklist was first and which checklists were subsequent, so for each of the 45 nurses who had more than one checklist completed, we averaged his or her checklist scores to create a single delayed post-test score and domain-specific scores. We analyzed checklists completed between May 2015 and September 2016.

### Patient Volume and Referrals

We abstracted data from health center patient registries, transfer forms and notes, and hospital records to identify the number of patients seen at the intervention health centers for breast concerns, the number evaluated at the hospital, and the appropriateness of referrals relative to the clinical algorithms.

### Statistical Analysis

We used Wilcoxon signed rank tests to compare nurses’ and CHWs’ pretraining scores to their scores on immediate post-tests or checklists and their scores on delayed post-tests or mentoring checklists. We used multivariable logistic regression to examine the association of CHW and nurse characteristics with the likelihood of having a score > 90% on immediate post-tests.

### Ethical Approval

Approval for this study was obtained from the Partners Healthcare Institutional Review Board in Boston, Massachusetts, and the Rwanda National Ethics Committee.

## RESULTS

### Nurses’ Knowledge

One hundred twenty-seven nurses participated in the early detection trainings; nurse characteristics are provided in Table 1. Nurses had a mean age of 36, and most (72.2%) were female. Only two nurses (1.6%) had any previous training in breast health, and nearly 30% had never heard of breast cancer. Before the trainings, nurses’ median score on the written knowledge assessment (proportion of 23 questions correct) was 73.9% (interquartile range [IQR], 69.6% to 82.6%; Table 2). Immediately after the trainings, the median overall score increased to 91.3% (IQR, 87.0% to 95.7%; *P* < .001). Three months after the training, the median delayed post-test score was 91.3% (IQR, 82.6% to 95.7% [*P* < .001] when delayed post-test scores were compared with pretest scores). Nurses’ scores on treatability of breast cancer and treatment availability were higher than scores on breast cancer risk and signs/symptoms.

**Table 1:**
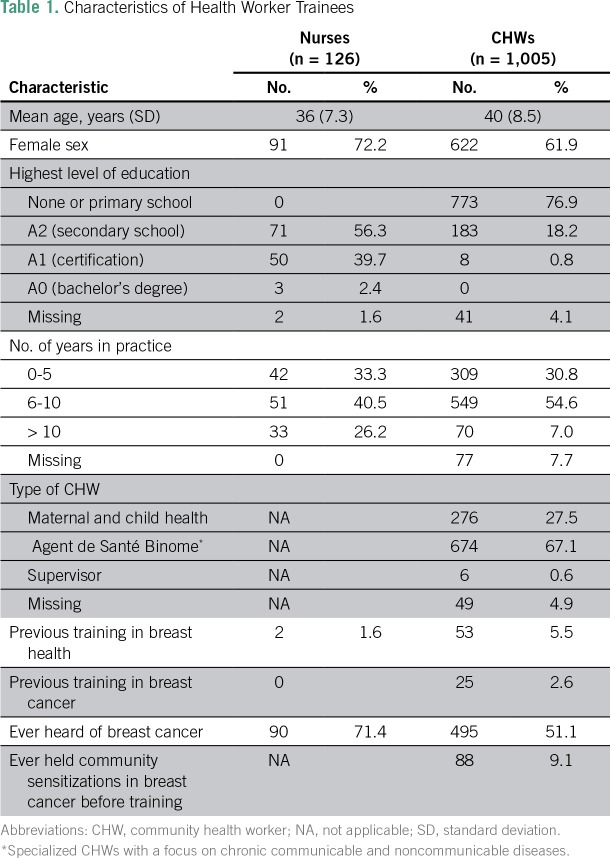
Characteristics of Health Worker Trainees

**Table 2:**
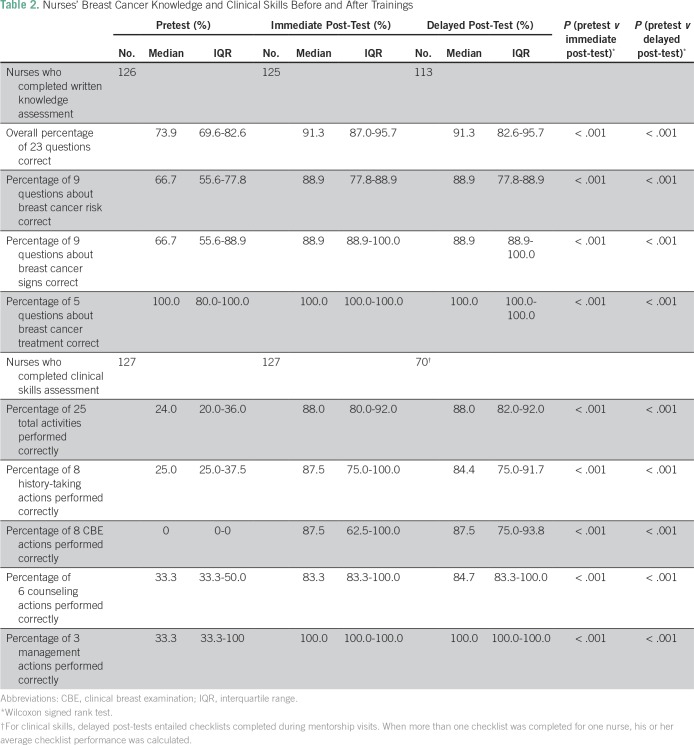
Nurses’ Breast Cancer Knowledge and Clinical Skills Before and After Trainings

### Nurses’ Skills in Evaluation and Management of Breast Concerns

Before the training, nurses’ median overall score (percentage of 25 actions performed correctly) on clinical skills checklists was 24% (IQR, 20.0% to 36.0%; Table 2). The median score increased to 88.0% (IQR, 80.0% to 92.0%) after the training (*P* < .001). During the mentorship phase, over 18 months, 157 checklists were administered by the nurse-midwife mentor who observed 70 trained nurses assigned to the breast clinics on days when she made mentoring visits. Among the 70 nurses, overall median checklist scores were 88.0% (IQR, 82.0% to 92.0%). Performance on the eight elements of the CBE section was particularly weak before the training (median, 0%) but CBE scores increased to 87.5% immediately after the training (*P* < .001) and remained at 87.5% during mentorship (*P* < .001).

### CHWs’ Knowledge

One thousand seventy-six CHWs participated in at least one nurse-led training; CHWs’ characteristics are provided in Table 1. Overall, 1,005 CHWs completed a pretest, 999 completed a post-test, and 946 completed a delayed post-test. Of the CHWs completing a pretest, 62% were female and 77% had had no formal education or only a primary school education. Approximately 5% reported previous training in breast health and about half had ever heard of breast cancer. Eighty-eight CHWs (9.1%) reported having previously led community education sessions about breast cancer. Before the trainings, CHWs had a median overall score (percentage of 16 questions correct) of 75% (IQR, 68.8% to 87.5%) on the written knowledge assessment (Table 3). Immediately after the trainings, the median overall score increased to 93.8% (IQR, 87.5% to 100.0%; *P* < .001). The median overall score on delayed post-tests was also 93.8% (87.5% to 100.0%; *P* < .001 for pretest *v* delayed post-test scores). Median baseline scores were 80.0%, 80.0%, and 83.3% on the groups of questions about breast cancer risk, signs and symptoms, and availability of treatment, respectively.

**Table 3:**
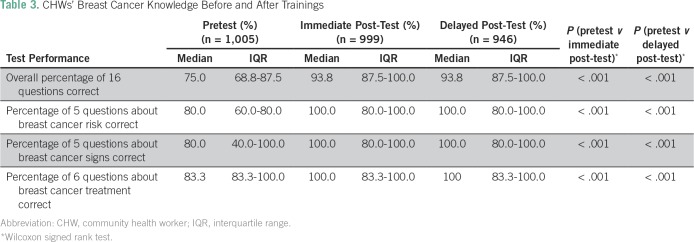
CHWs’ Breast Cancer Knowledge Before and After Trainings

### Factors Associated With Higher Scores on Post-Training Knowledge Tests

In multivariable logistic regression analyses that assessed scores on immediate post-tests, nurses who reported having heard of breast cancer at baseline were more likely to have a score of > 90% compared with nurses who had not heard of breast cancer, when adjusting for other factors (odds ratio [OR], 2.61; 95% CI, 1.14 to 5.98; Table 4). Other nurse characteristics were not significantly associated with higher scores. Among CHWs, those with more education were more likely to have a score of > 90% compared with less educated CHWs when adjusting for other factors (OR, 1.70; 95% CI, 1.17 to 2.47; Table 4). ASB CHWs were also more likely to have a score > 90% (OR, 1.82; 95% CI, 1.35 to 2.46). CHWs who reported having previously received training in breast health were less likely to have a score > 90% on the immediate post-test (OR, 0.52; 95% CI, 0.28 to 0.98).

**Table 4:**
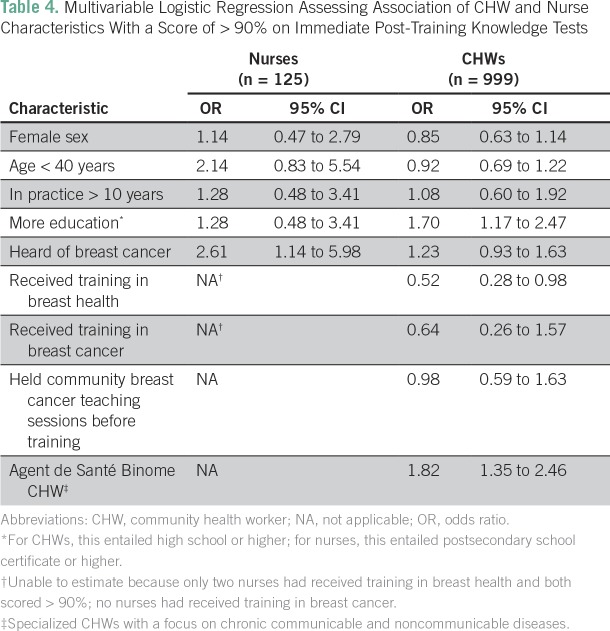
Multivariable Logistic Regression Assessing Association of CHW and Nurse Characteristics With a Score of > 90% on Immediate Post-Training Knowledge Tests

### Patient Care and Referrals

In delayed post-tests, 928 CHWs (98.1%) reported holding education sessions with their communities (*v* 9.1% before the training), and 924 CHWs (97.7%) reported speaking with individual clients about breast health. Interim review of health center and hospital records demonstrate that from May 2015 through March 2017, 1,560 patients were seen at the intervention health centers for breast concerns. Of these, 325 were referred to BCCOE and 260 were evaluated at the weekly breast clinic at BCCOE. Of those evaluated at the BCCOE clinic, 196 (75.4%) had a mass on examination, and an additional 34 (13.1%) had another abnormal finding on examination (eg, skin changes or nipple discharge); these patients were deemed to be appropriate for referral on the basis of the training algorithms developed for this study (Figs 1 and 2). Of the 30 patients with no concerning findings on examination, 20 were deemed to have been appropriately referred by receiving clinicians. Four patients (1.5% of those seen at the hospital) with breast pain were felt to have been potentially referred inappropriately (eg, they lacked documentation of failing conservative management), and for six patients (2.3%), the appropriateness of the referral was not possible to determine from clinical records. Overall, 88 patients (33.8% of those seen at the hospital) had biopsies, and 18 (6.9%) were diagnosed with cancer.

## DISCUSSION

This training intervention in Rwanda demonstrated that after relatively brief trainings, lay CHWs and rural primary care nurses learned key information about breast cancer signs, symptoms, and treatability, and this knowledge was sustained. Nurses’ skills in CBE and patient evaluation were notably weak before the training but improved substantially with practical training using breast models followed by regular clinical mentorship. Weekly breast clinics were successfully established in nurses’ rural health centers, and 96.1% of patients subsequently evaluated at the hospital were felt to have been appropriately referred.

Misunderstandings, myths, and stigma about breast health and breast cancer are common in LMICs, even among health providers.^4,14,15^ Our previous analysis of diagnostic delays at BCCOE^5^ suggested that primary health care providers were not making appropriate management decisions when evaluating patients with breast findings of concern. In this study, nurses’ pretest scores demonstrated that CBE skills were indeed inadequate. Also of concern, CHWs who had received prior trainings in breast health were actually less likely to perform well on our tests, suggesting that previous trainings were of questionable quality.

A small but growing body of literature describes efforts to train health workers in LMICs in early detection of breast cancer.^8,16-18^ Most of the literature focuses on screening asymptomatic women with CBE.^17,19-21^ In contrast, a central aspect of this project’s approach was its focus on expediting evaluation of breast symptoms. We chose to focus on symptomatic women because our early research on diagnostic delays suggested that much could be gained by reducing the time to diagnosis among women who already had symptoms. In addition, targeting symptomatic patients would optimize the positive predictive value of CBE and improve the project’s feasibility because it could result in lower patient volume than systematic screening and could help ensure that patients’ needs could be met. We developed a breast care delivery system that included weekly breast clinics at the health centers, clinical documentation forms and referral algorithms, and a hospital breast clinic to receive patients in need of additional evaluation.

We gathered feedback from nurse and CHW trainees and hospital clinician trainers to identify areas in which the training and project implementation were successful or needed modification. Nurses and the mentor noted the burden of adding an additional clinic and more patients to already busy health centers, particularly during the project’s initial phase when patient volume was highest. However, training all nurses in each health center was considered useful to ensure that any of the health center’s nurses could staff the breast clinic. Nurses also recommended more coordination and communication regarding transferred patients. To improve coordination between the hospital and health centers and support local leadership, we identified breast health champions at each health center who could serve as main contacts and advocates for the early detection program and facilitate communication about patients in need of follow-up. These champions are receiving additional hands-on training at BCCOE’s weekly breast clinic, with the goal of empowering them to be trainers at their health centers. Turnover of health care workers is often a significant issue in under-resourced health systems, so we have held additional trainings for nurses who started work after the program’s initiation.

Our study has some limitations. The chief limitation relates to the generalizability of our findings to the rest of Rwanda and other rural low-resource settings. Although nurses and CHWs in Burera District are likely to be typical of rural Rwandan health care workers, they may have had more baseline awareness of cancer and its treatability because of their proximity to BCCOE. Nevertheless, knowledge at baseline for nurses and CHWs in our study was quite low. In addition, although much of our training intervention is likely to be highly replicable in other districts and other low-income countries, we have benefited from trainers who are clinicians at BCCOE and who have extensive experience in diagnosing breast disease and breast cancer, experience not typically found in a district without a cancer facility. It will be important to examine the impact of similar trainings in regions without the resources available at BCCOE. An additional limitation of our analysis is that we examined the clinical presentations of only the 260 patients seen at the project’s weekly hospital breast clinic from May 2015 through March 2017. Sixty-five of the patients referred from intervention health centers during this period were not seen at the breast clinic; these may have been seen at BCCOE’s general oncology clinic, general outpatient clinic, or emergency room, whereas others may have been lost to follow-up. If these patients were less likely to have highly concerning breast symptoms, this analysis may overestimate the appropriateness of health care nurses’ referrals. Data collection from other hospital clinics is underway to permit analysis of the impact of the project on patients’ diagnoses, cancer stage, and loss to follow-up.

Our project suggests that with sustained clinical mentorship and support in developing breast health care delivery systems, rural health care workers can acquire the necessary knowledge to educate patients, effectively triage breast concerns, and make appropriate referral decisions. Additional analyses will identify the resources necessary to meet patients’ needs if this project were scaled up or expanded to include CBE screening. In addition, analysis of clinical outcomes will be critical to identify the value of this initiative in reducing patients’ delays and facilitating earlier-stage diagnoses. Given limited capacity at health centers, district hospitals, and tertiary care facilities to which patients are referred, we feel that expanding this initiative to screening of asymptomatic disease should be pursued only with adequate resources for staff training and support and when strong systems are in place for referrals for breast imaging, efficient tissue diagnosis, high-quality cancer treatment, and patient tracking to minimize loss to follow-up.In conclusion, our training initiative significantly improved the knowledge and skills of rural Rwandan CHWs and nurses regarding breast health and breast cancer and led to largely appropriate hospital referrals. Ongoing analyses will evaluate the impact of the training on clinical services, patient delays, and breast cancer stage at diagnosis.
